# Pediatric ectodermal dysplasia with immunodeficiency caused by a hemizygous *IKBKG* frameshift variant: a case report and literature review

**DOI:** 10.3389/fimmu.2026.1831594

**Published:** 2026-09-15

**Authors:** Yifan Xuan, Xin Chang, Yanfei Yang, Yanfen Wang

**Affiliations:** Shanxi Bethune Hospital (Shanxi Medical Academy), Third Hospital of Shanxi Medical University, Department of Pediatrics, Shanxi Bethune Hospital, Tongji Shanxi Hospital, Taiyuan, China

**Keywords:** children, ectodermal dysplasia with immunodeficiency, IKBKG, immunodeficiency, NF-κB

## Abstract

**Objective:**

This case report looks at a child’s ectodermal dysplasia with immunodeficiency (*EDA-ID*), caused by a mutation in the *IKBKG* gene, and other relevant cases.

**Methods:**

A pediatric patient diagnosed with *EDA-ID* was examined at Shanxi Bethune Hospital in July 2025. Clinical data were systematically collected, and whole-exome sequencing (WES) was performed on blood samples from the patient and their family members to identify potential pathogenic variants. Segregation analysis and a comprehensive literature review were performed.

**Results:**

The patient exhibited symptoms including fever, mucoid bloody stools, and atopic dermatitis. WES analysis revealed the presence of a hemizygous variant, designated c.1167dup (p. Glu390ArgfsTer5), within the *IKBKG* gene. In accordance with the guidelines established by the American College of Medical Genetics and Genomics (ACMG) and the Clinical Genomics Resource (ClinGen), this variant is classified as pathogenic (PVS1+PS4).

**Conclusion:**

In pediatric patients with early-onset findings, immunodeficiency should be considered after excluding infections, rheumatic or malignancies. Key clinical features that raise suspicion include sparse hair, reduced sweating, dry skin, recurrent infections, treatment-refractory atopic dermatitis, persistently elevated acute-phase inflammatory markers, and immune dysfunction. Genetic testing facilitates early diagnosis, thereby supporting treatment planning. In this study, we identified a pathogenic *IKBKG* variant (c.1167dupC) in a pediatric patient with *EDA-ID* via WES, expanding the current understanding of genotype-phenotype correlations.

## 1 Introduction

Ectodermal dysplasia with immunodeficiency (*EDA-ID*) is a condition resulting from impaired nuclear factor κB (NF-κB) signaling due to genetic mutations or associated defects. Its clinical presentation is diverse and includes ectodermal dysplasia, lymphedema, osteopetrosis, and multiple immunological abnormalities. Patients may experience recurrent infections, diminished antibody response to polysaccharides, hypogammaglobulinemia, hyper-IgM syndrome, impaired natural killer (NK) cell cytotoxicity, and autoimmune disorders (1). Differential diagnoses include hyper-IgM syndromes, common variable immunodeficiency(CVID), and other combined immunodeficiencies. Diagnosis relies on clinical manifestations, immunological phenotype, and genetic testing. Two genes associated with *EDA-ID* have been identified: mutations in the *IKBKG* gene, which encodes the NF-κB essential modulator (*NEMO*), follow an X-linked inheritance pattern (2); and mutations in the NFKBIA gene, encoding the NF-κB inhibitor α (IκBα), cause autosomal *EDA-ID* (3). X-linked EDA-ID *(XL-EDA-ID)* is a relatively uncommon condition. The c.1167dup mutation in *IKBKG* has been previously reported; however, the clinical phenotype associated with this recurrent variant is not fully characterized. This report describes a pediatric patient with *EDA-ID* caused by the *IKBKG* c.1167dup mutation, further expanding the clinical phenotype associated with this variant. By combining a literature review with this case study, we aim to enhance clinicians’ awareness of this condition and improve early diagnosis.

## 2 Objectives and methods

A four-month-old male infant was admitted to our hospital due to recurrent leukocytosis and bloody stools. After obtaining informed consent from the family, we collected clinical data and drew 5 mL of peripheral blood samples from both the infant and his parents for genetic analysis. We also collected clinical information, including medical history, family history, laboratory findings, imaging studies, and treatment records. A comprehensive literature search was performed using the keywords “ectodermal dysplasia with immunodeficiency”, “X-linked ectodermal dysplasia with immunodeficiency”, “*NEMO-ID*”, and “*IKBKG* gene” in CNKI, Wanfang Database, VIP Database, and PubMed from database inception to August 31, 2025. Inclusion criteria were:(1)confirmed *IKBKG* mutation;(2)clinical diagnosis of EDA-ID, OL-EDA-ID (Osteopetrosis, Lymphedema, Anhidrotic Ectodermal Dysplasia with Immunodeficiency), or isolated immunodeficiency;(3)available clinical or immunological data;(4)age ≤18 years. Only articles published in English or Chinese were included. Exclusion criteria were duplicate publications or lack of original data. The literature screening was conducted in strict accordance with the PRISMA guidelines, and the specific screening procedure is presented in **Figure 1** (PRISMA flow diagram).

**Figure 1:**
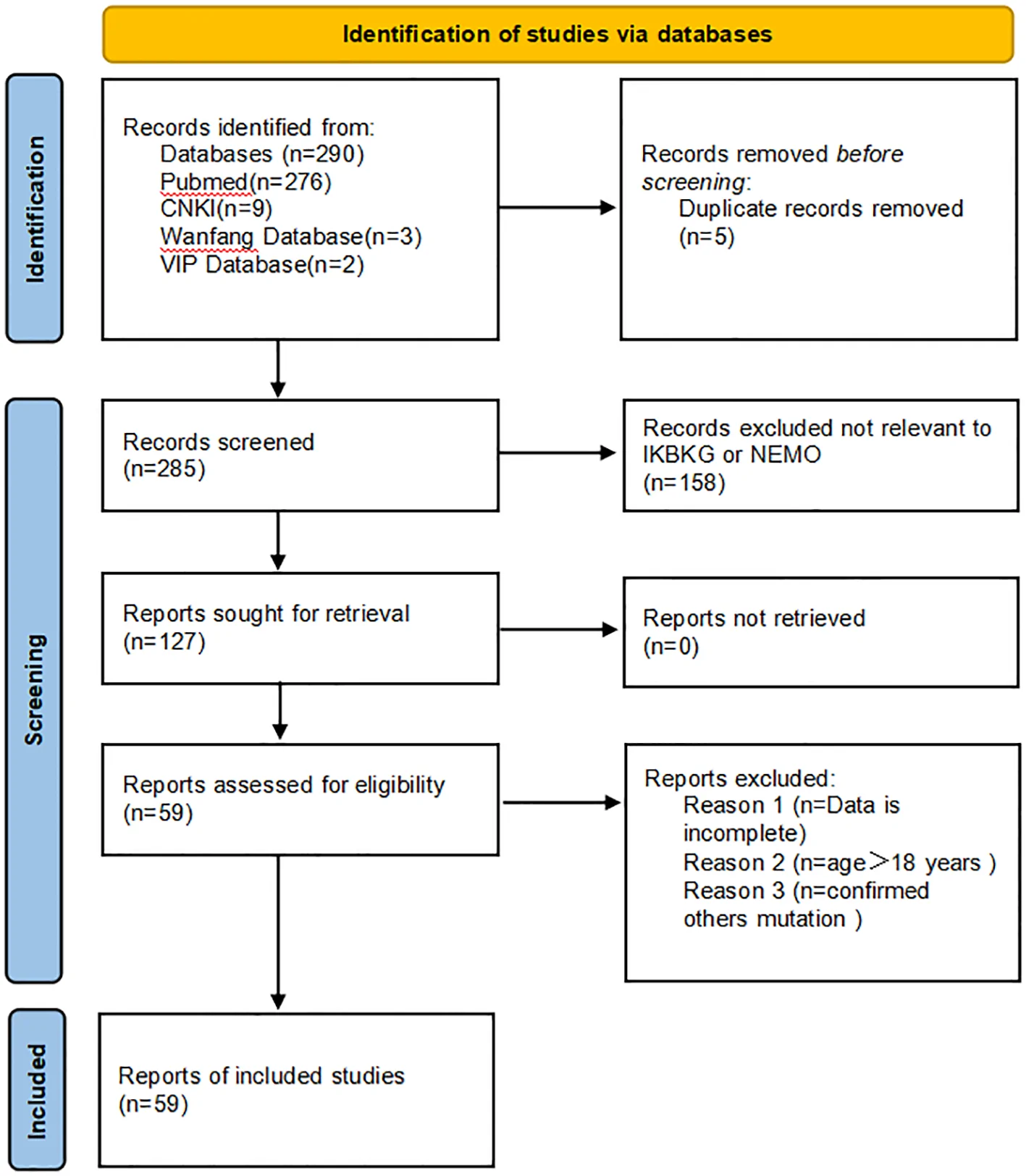
PRISMA flow diagram.

## 3 Case data and literature findings

### 3.1 Case presentation

Family and personal history: Non-consanguineous parents, no history of infections or infant deaths. The patient was the first-born at 40^+6^ weeks of gestation; oligohydramnios was noted at 37 weeks but not addressed. Birth was complicated by grade II amniotic fluid contamination and ecchymoses; platelets and coagulation were normal. The infant received antimicrobial and hemostatic therapy and was discharged. At 7 days of life, eczema developed that was unresponsive to topical therapy. Umbilical cord detachment occurred at 45 days with minor discharge. No perianal abscess was noted. The newborn has completed the screening for four conditions (Phenylketonuria, Congenital Hypothyroidism, Congenital Adrenal Hyperplasia, Glucose-6-phosphate dehydrogenase deficiency) and HIV nucleic acid test was normal.

The infant presented with diarrhea at two months of age, with 7 to 10 episodes per day; the stools contained mucus and bloody pus. A thorough stool examination revealed the presence of 3 to 5 red blood cells (RBCs) per high-power field (HPF), along with 10 to 20 white blood cells (WBCs) per HPF. Further investigation was prompted by the patient’s elevated white blood cell count, which exhibited fluctuations and was measured at a range of 15.75-29.53 x 10^9^/L. Given the infant’s concurrent presentation of intractable eczema, the probable etiology was identified as a food protein allergy. Consequently, the therapeutic regimen was modified to encompass lactase, montmorillonite, probiotics, and an amino acid formula. However, the child’s diarrhea showed no improvement, and routine stool examinations continued to– reveal the presence of red and white blood cells. Consequently, the child was admitted to our hospital for treatment at 4 months of age. Upon admission, the patient was examined physically, revealing a height of 68 centimeters, a weight of 6 kilograms, and a BMI of 13.0kg/m^2^ (3rd–5th percentile by WHO child growth standards). The anterior fontanelle was observed to be flat and soft, with dimensions measuring approximately 1.5 × 1.5 cm. The subject’s scalp hair and eyebrows were sparse, with no other body hair present. Teeth had not yet erupted, and nails appeared normal. In the course of febrile episodes, the occurrence of sweating was reduced or ceased. The rash was characterized by its diffuse appearance, non-pruritic, and a pale red coloration. It was also noted that the rash exhibited a circular configuration and was located in a superficial layer of the skin. Cardiac, pulmonary, and abdominal examinations revealed no abnormalities. The perianal region exhibited no signs of redness, swelling, or ulceration. Post-admission laboratory monitoring revealed an elevated white blood cell count, with red and white blood cells visible in routine stool examinations. Further diagnostic procedures, including stool culture, did not reveal any signs of a specific infection. Normal platelet and coagulation function levels suggest the absence of coagulation disorders for the time being. Abdominal X-ray and ultrasound revealed no congenital intestinal anomalies. The most probable diagnosis was that of infectious diarrhea, and the child was treated with cefotaxime for the infection. The child’s diarrhea exhibited a marginal improvement, with red and white blood cells in the stool returning to normal levels. However, the fecal occult blood test remained positive. The child developed a fever and diarrhea after contact with a sick family member. Inflammatory bowel disease and immunodeficiency were considered as possible causes. Comprehensive respiratory NGS testing revealed Pneumocystis jirovecii pneumonia and parainfluenza virus type 3 infection. The treatment regimen was adjusted to penicillin combined with cefoperazone/sulbactam. Monitoring of immune function revealed hypogammaglobulinaemia, suggesting immunodeficiency, and the patient was transferred to Beijing Children’s Hospital. During hospitalization, the patient underwent bone marrow aspiration, lumbar puncture, bronchoscopic alveolar lavage and genetic testing. The patient was placed on mechanical ventilation and treated with trimethoprim-sulfamethoxazole for infection and intravenous immunoglobulin (IVIG). Genetic testing results confirmed *EDA-ID* caused by a mutation in the *IKBKG* gene. In January 2026, the patient underwent allogeneic hematopoietic stem cell transplantation. Currently, the patient is in good health with no post-transplant complications reported, and further evaluation is scheduled for July 2026.

### 3.2 Laboratory testing

#### 3.2.1 Routine stool examination and occult blood test (March 18-July 28, 2025)

The specific details are provided in **Tables 1** and **2**.

**Table 1:** Dynamic Changes in Routine Stool Parameters.

Times	Fecal occult blood	Leukocytes/HP	Red blood cells/HP
2025-07-20	+	20-30	1-3
2025-07-22	+	5-10	3-5
2025-07-23	+	5-10	1-3
2025-07-24	+-	5-10	0-1
2025-07-25	+	0-1	5-10
2025-07-28	+	0	0

**Table 2:** Dynamic Changes in Blood Cell Analysis and C-Reactive Protein.

Times	WBC*10^9^/L	neutrophil%	hemoglobin(g/l)	Platelets*10^9^/L	C-Reactive Protein (mg/l)
2025-03-18	29.53	70.0%	190	342	<0.5
2025-03-23	20.50	26.2%	182	253	<0.5
2025-04-09	15.75	22.7%	120	334	<1.0
2025-04-28	16.52	18%	105	377	15.2
2025-07-19	26.20	35.2%	109	512	13.17
2025-07-22	29.70	42.1%	108	526	15.53
2025-07-24	22.22	44.0%	120	166	4.97
2025-07-28	42.99	43.5%	116	275	8.34

Immunology: Immunoglobulin IgG levels were significantly reduced, while IgA, IgM, and IgE remained within normal ranges; cytokine IL-17 was elevated (47.254 pg/ml). Lymphocyte subset analysis revealed a decreased proportion of CD8+T cells and an increased proportion of B cells. Detailed data are provided in **Table 3**.

**Table 3:** Specific lymphocyte subset data.

	Outcome	Reference Range
Percentage of total T-Cells (CD3+CD45+)	57.3%	65%-79%
Absolute total T-cell count (CD3+CD45+)	7062/ul	650-1880/ul
Percentage of Th cells (CD3+CD4+)	46.6%	34%-52%
Absolute Th cell count (CD3+CD4+)	5745/ul	340-1072/ul
Percentage of CD8+T cells (CD3+CD8+)	10.2%	21%-39%
Absolute CD8+T cells count (CD3+CD8+)	1243/ul	210-742/ul
Th/CD8+T cells	4.61	1-2
Percentage of B cells (CD3–CD19+)	30.2%	9.02%-14.2%
Absolute B cell count (CD3–CD19+)	3432/ul	90-660/ul
Percentage of NK cells (CD3-CD56+)	6.6%	4.6%-16.0%
Absolute NK cell count(CD3–CD56+)	749/ul	46-590/ul
Percentage of NK-like T cells(CD3+CD56+)	0.9%	0%-12%
Absolute NK-like T cell count (CD3+CD56+)	107/ul	
Immunoglobulin A	0.13g/l	0.7-4.0g/l
Immunoglobulin M	0.74g/l	0.4-2.3g/l
Immunoglobulin G	0.58g/l	7.0-16.0g/l
Immunoglobulin E	11.8IU/ml	≤15IU/ml

Pathogens:

- Parainfluenza virus type 3(by tNGS and respiratory panel)
- Pneumocystis jirovecii(by blood and bronchoalveolar lavage tNGS)
- Throat swab: Enterococcus faecalis(small amount)
- Stool culture: negative
- Blood culture: negative
- Acid-fast bacilli (AFB): negative

Imaging: Abdominal X-ray showed intestinal gas; abdominal and intestinal ultrasound normal; cardiac ultrasound showed patent foramen ovale at birth, closed on follow-up.

Peripheral smear: The morphology of red blood cells varies in size, with some exhibiting enlarged pale staining areas. In terms of white blood cell morphology, certain types of neutrophils have been observed to contain toxic granules in their cytoplasm. Regarding platelet morphology, there were observations of platelet aggregation and elevated platelet counts. The total cell count was 100 cells.

Bone marrow: The morphology of red blood cells varies in size, with some exhibiting enlarged pale staining areas. In terms of white blood cell morphology, certain types of neutrophils have been observed to contain toxic granules in their cytoplasm. Regarding platelet morphology, there were observations of platelet aggregation and elevated platelet counts. The total cell count was 100 cells.

#### 3.2.2 Whole-exome sequencing

WES revealed a hemizygous frameshift mutation in *IKBKG* (c.1167dup; p.Glu390ArgfsTer5), confirmed by Sanger sequencing: the patient is hemizygous, the mother is a heterozygous carrier, and the father is wild type. This mutation leads to premature translation termination and loss of protein function (PVS1). It has been reported in multiple *EDA-ID* patients (PS4), is extremely rare in population databases, and follows an X-linked recessive pattern in the family. According to ACMG guidelines, the variant is classified as pathogenic (PVS1+PS4).

In addition, the patient carries a heterozygous pathogenic variant in *DUOX2*(c.3250C>T; p.Arg1084Ter), inherited from the father (mother wild type). This variant is associated with thyroid secretory disorder type 6(autosomal recessive). It is likely incidental, as no thyroid dysfunction was observed.

Limitation: No functional validation(e.g. NF-κB phosphorylation, IκBα degradation, TNF-α stimulation assays)was performed due to the unavailability of frozen samples. The detailed genetic testing results and the Sanger sequencing chromatograms are shown in **Table 4** and **Figure 2**, respectively.

**Table 4:** WES genetic testing results for pediatric patients.

Genes	Chromosome	Nucleotide variation in transcript numbering	Genotype	Pathogenic classification	Mode of inheritance
*IKBKG*	chrX:153792583-153792584	exon10 c.1167dup	Hemizygous Father: Wild-type Mother: Heterozygous	pathogenic	X-linked inheritance
*DUOX2*	chr15:45392025	exon25 c.3250C>T	Heterozygous Father: heterozygous Mother: wild-type	pathogenic	Autosomal Recessive

*IKBKG*(OMIM:300248); *DUOX2*(OMIM:606759).

**Figure 2:**
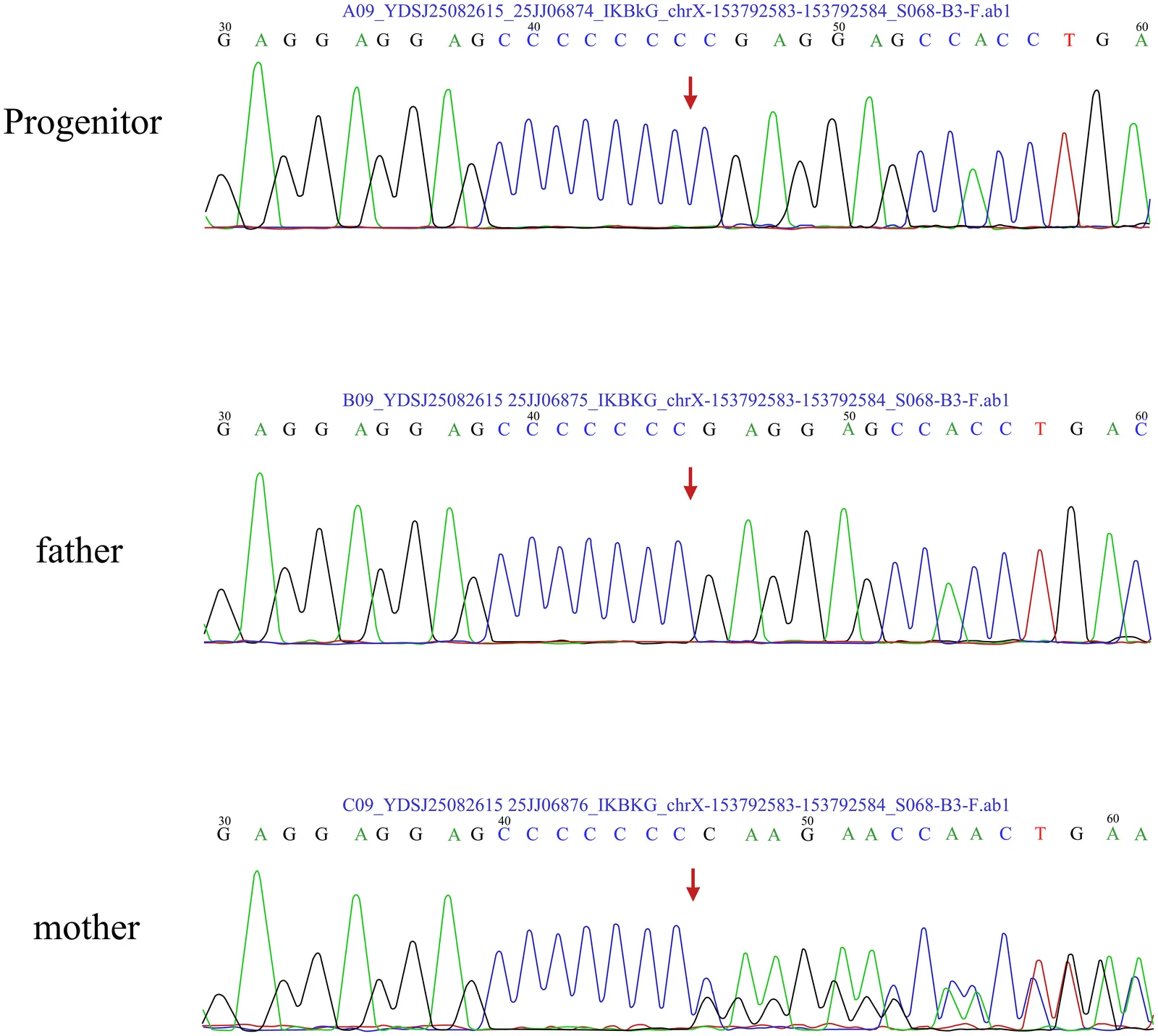
Sequencing map of the *IKBKG* gene in each member of the *EDA-ID* family. Pediatric patient (hemizygous); Mother (heterozygous); Father (wild-type). Arrows indicate variant sites.

### 3.3 Literature review

The comprehensive literature search identified 56 English-language and 3 Chinese-language publications, reporting 65 *EDA-ID* patients with *IKBKG* variants (excluding the present case). Among these, eight cases were identified as *OL-EDA-ID*, and 11 cases were classified as the non-ectodermal dysplasia subtype, which is characterized by pure immunodeficiency. The c.1167dup mutation identified in this case is one of the most frequently reported pathogenic mutations to date. The detailed list of included references is provided in **Table 5**.

**Table 5:** Clinical manifestations, immunological phenotypes, treatment and prognosis of diseases associated with the *IKBKG* gene.

Cases	Nucleotide or amino acid alterations	Family history	EDA	Infectious diseases	Immunological phenotypes	Outcome
1 (4)	c.1117 + 5G>C	+	–	+	IgA↓, IgG↓	Death
2 (5)	c.111dupC(p.Met38fsTer48)	–	–	+	IgA↓, IgG↓, IgM↑	Death
3 (6)	c.1056-1G>A	–	–	+	NK cells↓	IVIG
4 (7)	c.601C>T (p.Gln201Ter, Q201X)	+	–	+	–	Death
5 (8)	c.931C>G (p.Asp311Glu, D311E)	–	–	+	–	HSCT
6 (9)	c.956G>A (p.Arg319Gln, R319Q)	–	–	+	IgG↑	Treatment for tuberculosis
7 (10)	c.1238A>G (p.His413Arg, H413R)	–	+	+	All IgG subclasses↓, B cells↓, NK cells↓	Death
8 (11)	c.1259A>G (p.Ter420Trp, X420W)	+	+	+	Impaired TNF-α response	Death
9 (12)	c.1259A>G (p.Ter420Trp, X420W)	+	+	+	IgG↓, T cell deficiency	Death
10 (13)	c.1259A>G (p.Ter420Trp, X420W)	+	+	+	IgG↓, lack of antibodies to polysaccharide antigens	Death
11 (14)	c.1259A>G (p.Ter420Trp, X420W)	+	+	+	Impaired TNF-α response	Death
12 (15)	c.1167dupC (p.Glu390fs, E390fs)	+	+	+	All IgG subclasses↓, CD3+ T cells↑, CD4+ T cells↑, NK cells↓	HSCT
13 (16)	c.1167dupC (p.Glu390fs, E390fs)	+	+	+	IgG↓, NK cell activity↓, B cells↑	HSCT
14 (17)	c.1167dupC (p.Glu390fs, E390fs)	+	+	Unknown	–	Unknown
15 (18)	c.1167dupC (p.Glu390fs, E390fs)	+	+	*+*	IgA↑, production of polysaccharide-specific antibodies↓	Unknown
16 (19)	c.169G>A (p.Glu57Lys, E57K)	–	–	+	IgG↓, specific polysaccharide antibody deficiency, B cells↓	IVIG
17 (20)	c.1167dupC (p.Glu390fs, E390fs)	+	+	+	IgG↓	HSCT
18 (21)	c.1167dupC (p.Glu390fs, E390fs)	+	+	+	IgG↓	HSCT
19 (21)	c.1167dupC (p.Glu390fs, E390fs)	–	+	Unknown	IgG↓, NK cells↓	Death
20 (21)	c.1167dupC (p.Glu390fs, E390fs)	–	+	+	IgG↓, CD8+ T cell proportion↓, NK cells↓	Death
21 (18)	c.1250G>T (p.Cys417Phe, C417F)	–	+	+	IgA↑, IgM↑, IgG↓, production of polysaccharide-specific antibodies↓	IVIG
22 (22)	c.1171G>A (p.Arg352SerfsTer373, R352Sfs*373)	–	+	+	IgG↓, IgM↑	HSCT
23 (23)	c.1027 + 5G>A	+	+	+	IgG↑, IgA↓, IgM↓, T cells↑, B cells↓	Unknown
24 (24)	c.944A>C (p.Glu315Ala, E315A)	–	+	+	IgG↑, T cells↑	Death
25 (25)	c.916G>A (p.Asp306Asn, D306N)	+	+	+	IgA↑	Death
26 (26)	c.1249T>C (p.Cys417Arg, C417R)	–	+	+	IgG↓	IVIG
27 (18)	c.1249T>C (p.Cys417Arg, C417R)	–	+	+	IgG↓, IgA↓, IgM↑	Death
28 (18)	c.1249T>C (p.Cys417Arg, C417R)	–	+	+	IgG↓, NK cells↓	IVIG
29 (27)	c.1171G>T (p.Glu391Ter, E391X)	–	+	+	T cells↓, B cells↑	IVIG
30 (27)	c.1171G>T (p.Glu391Ter, E391X)	–	+	+	T cells↓, B cells↑	IVIG
31 (27)	c.1171G>T (p.Glu391Ter, E391X)	–	+	+	T cells↓, B cells↑	IVIG
32 (24)	c.761G>A(p.Arg254Gln, R254Q)	–	–	+	IgA↓, IgG↓, B cells↓, CD4+ T cells↓	Death
33 (28)	c.1117 + 1G>A	–	–	+	IgA↑, IgM↓, memory B cells↓	Death
34 (27)	c.1171G>T (p.Glu391Ter, E391X)	–	+	+	T cells↓, B cells↑	IVIG
35 (18)	c.1171G>T (p.Glu391Ter, E391X)	–	+	+	IgA↓, IgG↓, IgM↑	Death
36 (29)	c.458T>G (p.Leu153Arg, L153R)	–	+	+	NK cell cytotoxicity deficiency	HSCT
37 (18)	c.1171G>T (p.Glu391Ter, E391X)	–	+	+	IgA↓, IgG↓, IgM↑	Death
38 (30)	c.399 + 850_674dup (Dupfrom intron 3 to exon 6)	+	+	+	IgG↑, IgA↑, IgM↓, CD4+ T cells↓	Death
39 (30)	c.399 + 850_674dup (Dupfrom intron 3 to exon 6)	+	+	+	T cell proliferation↓	Death
40 (24)	Rearrangement from Intron 4-8	–	+	+	All Ig subclasses↓	Unknown
41 (24)	c.597G>A (A174_K224del)	–	+	+	IgA↓, IgG↓, IgM↑	Unknown
42 (31)	c.74_77delACGT(p.His25ArgfsTer14)	–	–	+	IgA↓, IgG↓, IgM↑	Unknown
43 (32)	c.613C>T (p.Gln205Ter, Q205X)	+	+	+	IgG↑, CD3+ T cells↓, CD4+ T cells↓, T cell proliferation↓	HSCT
44 (33)	c.505G>C (p.Ala169Pro, A169P)	–	+	+	T cells↓, B cells↑	Fusiximab
45 (24)	c.936C>G (p.Phe312Leu, F312L)	–	+	+	T and B cell ↓	Death
46 (34)	c.811_828del (p.Glu271_Ala276del)	–	+	+	IgG↑	Unknown
47 (34)	c.239T>C (p.Leu80Pro, L80P)	–	+	+	IgG↓, T cell proliferation↓	IVIG
48 (35)	c.518 + 866C>T	–	+	+	HLA-DR and CD4+ T cell ratio↑	Death
49 (36)	c.437T>G (p.Val146Gly, V146G)	–	+	+	IgG↓, IgM↓, IgA↑, CD8+ T cells↓, NK cells↓, B cells↓	Unknown
50 (37)	c.262_264delGAG(p.Glu88del, E88del)	+	+	+	IgG↓	Unknown
51 (38)	c.1167dupC (p.Glu390fs, E390fs)	+	+	+	IgM↑, IgE absent, IgA↓, T cells↑, TREC↓, NK cytotoxicity↓	Unknown
52 (39)	c.1056-1G>A(p.353-373)	–	–	+	B cell proliferation↓	Unknown
53 (40)	c.470A>C (p.Gln157Pro, Q157P)	–	+	+	IgG↓, T and B cell dysfunction	HSCT
54 (41)	c.1249T>C (p.Cys417Arg, C417R)	–	+	+	IgG↓	IVIG
55 (42)	c.769-1G>C	–	+	+	CD19+ B cells↓	Unknown
56 (43)	c.931G>A(p.Asp311Asn, D311N)	–	+	+	IgG↓, IgA↑	Unknown
57 (44)	c.337G>A (p.Asp113Asn, D113N)	–	+	+	IgE↑	Alive
58 (22)	c.337G>A (p.Asp113Asn, D113N)	–	+	+	production of polysaccharide-specific antibodies↓	IVIG
59 (6)	c.458T>G (p.Leu153Arg, L153R)	+	+	+	IgG↓, NK cells↓	IVIG
60 (45)	c.545G>C (p.Arg182Pro)	–	+	+	B cells↑	Unknown
61 (46)	c.490A>C (p.Thr164Pro, T164P)	+	+	+	IgM↑, T cells↓, B cells↓, NK cells↓	Death
62 (47)	c.667_669del(p.Glu223del)	–	+	+	IgM↓, IgG↑, NK cytotoxicity↓	IVIG
63 (47)	c.667G>A	–	+	+	IgM↓, B cell ↓	IVIG
64 (48)	c.932A>G (p.Asp311Gly, D311G)	–	+	+	anti-B heterologous hemagglutinin↓	Unknown
65 (49)	c.519-23A>T	–	+	+	IgG↓, IgM↓, IgA↑, CD4+ T cells↓	Death
66 (44)	c.169G>A (p.Glu57Lys, E57K)	–	+	+	IgG↓, IgA↓, CD4+ T cells↓	IVIG
67 (50)	c.768 + 5G>A	–	+	+	IgG↓, IgM↑	HSCT
68 (51)	c.520_523dupCAGG	+	+	+	IgM↓, CD3+ T cells↓, T cell proliferation↓	Death
69 (52)	c.445T>G (p.Cys149Gly)	–	+	+	Ig subclasses↓, CD4+/CD8+ ratio↑	Unknown
70 (4)	c.1241T>A	–	+	+	IgA↓, IgM↓	Death
71 (53)	c.1249T>G (p.Cys417Gly, C417G)	–	+	+	IgG↓, CD8+ T cells↓, B cells↓	IVIG
72 (54)	c.1167dupC (p.Glu390fs, E390fs)	–	+	+	IgG↓	HSCT
73 (49)	c.519-23A>T	–	+	+	IgG↓, IgM↓, IgA↑, CD4+ T cells↓	Death
74 (6)	c.1207C>T (p.Gln403Ter, Q403X)	–	+	+	IgG↓, CD3+ T cells↓, CD4+ T cells↓, NK cells↓	IVIG
75 (6)	c.1250G>A (p.Cys417Tyr, C417Y)	–	+	+	IgG↓, NK cells↓	IVIG
76 (55)	c.943G>C (p.Glu315Gln, E315Q)	–	–	+	CD4+/CD8+ ratio↑, NK cell activity↓	HSCT
77 (56)	c.517C>G (p.Arg173Gly, R173G)	–	+	+	Impaired vaccine antibody response	IVIG
78 (9)	c.944A>C (p.Glu315Ala, E315A)	+	+	+	CD40-dependent IL-12-induced selective injury	Death
79 (57)	c.1238A>C (p.His413Pro, H413P)	+	+	+	IgM↑, no pneumococcal antibodies	Death
80 (58)	c.1182_1183delTT(p.Phe394HisfsTer18)	+	+	+	IgM↑, IgG↓, B cells↑	IVIG
81 (44)	c.518 + 5G>A	+	+	+	IgG↓, IgM↑, memory B cells↓	Death
82 (59)	c.1167dupC (p.Glu390fs, E390fs)	–	+	+	IgA↓, IgM↓	Death
83 (60)	c.1167dupC (p.Glu390fs, E390fs)	+	+	+	–	Death
84 (9)	c.956G>A (p.Arg319Gln, R319Q)	–	+	+	CD40-dependent IL-12-induced selective injury	Treatment for tuberculosis

Cases 1–11 are classified as ID, cases 12–19 as OL-EDA-ID (OMIM:300301), and cases 20–84 as EDA-ID (OMIM:300291).

IVIG, intravenous injection Intravenous Immunoglobulin.

HSCT, Hematopoietic Stem Cell Transplantation. ↑ indicates an increase, while ↓ indicates a decrease.

The distribution of mutation types is summarized in **Table 6**. Frameshift or nonsense mutations were observed in the majority of cases, with common mutation sites including c.74-77delACGT, c.111dupC, c.1167dupC, c.1171G>T, c.1182-1183delTT, and c.520-523dupCAGG. Splicing site mutations were identified in cases with mutations such as c.1117 + 1G>A, c.1056-1G>A, c.1027 + 5G>A, c.769-1G>C, and c.768 + 5G>A. Missense mutations were frequently reported, involving amino acid substitutions including E57K, D311E, E315Q, R319Q, H413R, C417F, D306N, R254Q, A169P, V146G, D113N, L153R, F312L, L80P, R182P, T164P, D311G, D311N, C417R, C417Y, C417G, H413P, and R173G. Large-scale gene duplications or rearrangements, such as intron 3–exon 6 repeat and intron 4–8 rearrangement, were also documented.

**Table 6:** Distribution of mutation types.

Mutation type	Case number	Main mutation sites
Frameshift/Nonsense mutation	2, 5, 11, 14, 16, 24, 31–36, 40–47, 55, 60–62, 80	c.74_77delACGT; c.111dupC; c.601C>T; c.1182_1183delTT; c.1167dupC; c.1171G>A; c.613C>T; c.1171G>T; c.520_523dupCAGG; c.1207C>T
Splicing site mutation	6, 7, 9, 12, 25, 54, 68, 70, 77–79	c.1117 + 1G>A; c.1056-1G>A; c.1117 + 5G>C; c.518 + 5G>A; c.1027 + 5G>A; c.518 + 866C>T; c.769-1G>C; c.519-23A>T; c.768 + 5G>A
missense mutation	1, 3, 8, 10, 13, 15, 17–23, 26, 27, 37–39, 53, 54, 57, 63, 67, 69, 71–73, 75, 76, 81–84	E57K; D311E; E315Q; R319Q; H413R; Q157P; C417F; E315A; D306N; R254Q; A169P; V146G; C417R; D113N; L153R; F312L; L80P; D311N; R182P; T164P; c.667G>A; D311G; C149G; c.1241T>A; C417G; H413P; C417Y; R173G
Large-scale gene duplications/rearrangements	51, 64, 65	c.399 + 850_674dup (duplication from intron 3 to exon 6); rearrangement from intron 4 to intron 8
In-frame deletion	52, 58, 66, 74	c.262_264delGAG; c.597G>A (p.Ala174_Lys224del); c.811_828del; c.667_669del
Loss-of-stop variant	4, 28–30	c.1259A>G

The common immunological phenotypes are presented in **Table 7**. Decreased IgG levels were the most frequent finding, occurring in 40.5% of cases. Decreased IgA and elevated IgM were observed in 16.7% and 14.3% of patients, respectively. Reduced B-cell counts were noted in 10.7% of cases, while decreased NK cell counts or activity was reported in 15.5%. T-cell depletion or abnormal T-cell proportions were present in 20.2% of patients. A defect in specific polysaccharide antibody response was observed in 6.0% of cases, and an elevated CD4+/CD8+ ratio was identified in 2.4%.

**Table 7:** Common manifestations of immunological phenotypes.

Immunological phenotypes	Frequency	Case number
Decreased IgG	46.4%	1, 2, 5, 8–11, 13–15, 23–25, 27–29, 33, 34, 37–41, 43–47, 50, 52, 67, 69, 71, 77–79, 82
Decreased IgA	15.5%	5, 9–11, 27, 31, 38, 40–42, 48, 50, 52
Elevated IgM	16.7%	2, 5, 11, 24, 25, 37, 40–42, 52, 73, 79, 83
Decreased B-cell	15.5%	6, 7, 22, 25, 48, 50, 53, 57, 70, 73, 75, 82
Decreased NK cells/reduced activity	13.1%	12–15, 22, 33, 39, 46, 47, 57
T-cell depletion or abnormal proportion	29.8%	14, 27, 28, 33, 42, 47–50, 55–57, 59–62, 64, 65, 67, 68, 73, 77, 78, 80–82
Defect in the specific polysaccharide antibody response	6.0%	3, 8, 29, 36, 37
Elevated CD4+/CD8+ ratio	2.4%	18, 81

## 4 Discussion

X-linked ectodermal dysplasia with immunodeficiency (*XL-EDA-ID*) is caused by mutations in the *IKBKG* gene, which encodes the NF-κB essential modulator(*NEMO*) (1). Due to variations in gene mutation sites and their impact on different signaling pathways, the relationship between genotype and phenotype remains unclear. Only 77% of cases present as *EDA-ID*; the remainder present as immunodeficiency without ectodermal dysplasia (*ID*), or as more severe phenotypes (*OL-EDA-ID*) (2).

The *IKBKG* gene encodes *NEMO* (IKKγ), the regulatory subunit of the IKK complex, which is essential for the activation of NF-κB. The *NEMO* function is subject to regulation by phosphorylation and ubiquitination. Upon upstream signaling, IKK-mediated phosphorylation of IκBα promotes the translocation of NF-κB into the cell nucleus, thereby regulating biological processes such as immune responses, inflammatory processes, and cell survival (3). In addition, it is negatively regulated by three main classes of deubiquitinating enzyme: CyLD, A20 and OTULIN. CyLD has been shown to hydrolyze both linear and K63-linked ubiquitin chains, and by deubiquitinating *NEMO*, it inhibits TCR, BCR, and TLR-mediated canonical NF-κB activation, thereby regulating immune responses (61). A20 is involved in the negative regulation of various pro-inflammatory signals (such as TNF, IL-1, LPS, and the activation of T-cell and B-cell receptors). Typically, these signals activate NF-κB, and A20 acts to inhibit the excessive activation of NF-κB. A20 removes K63-linked polyubiquitin chains from proteins in NF-κB signaling pathways, preventing signal transmission and NF-κB activation (62, 63). OTULIN is a deubiquitinating enzyme which specifically cleaves linear polyubiquitin chains that are linked by N-terminal methionine residues and which are generated by the LUBAC complex. In patient cells, reduced levels of OTULIN protein and deubiquitinase activity lead to impaired deubiquitination of *NEMO* following TNF stimulation. This results in sustained IKK activity and IκBα phosphorylation, thereby negatively regulating NF-κB signaling (64, 65). A schematic diagram of the proposed mechanisms is presented in **Figure 3**.

**Figure 3:**
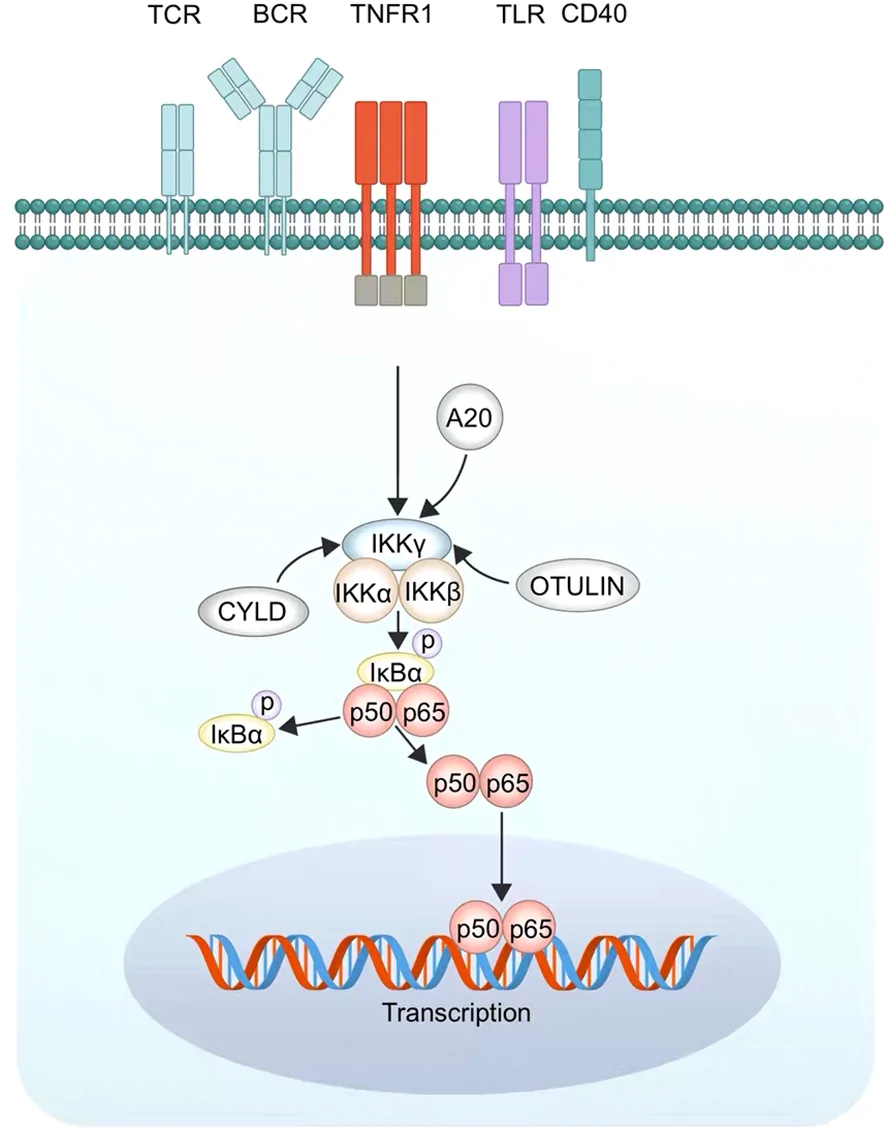
Activation and negative regulation of NEMO: Phosphorylation, Ubiquitination, and the Roles of Three Deubiquitinating Enzymes (CyLD, A20, OTULIN). Normally, NEMO (IKKγ) enables IKK-mediated IκBα phosphorylation and NF-κB activation, which drives expression of survival and immune genes. Deubiquitinases (CYLD, A20, OTULIN) remove ubiquitin chains from NEMO to terminate signaling. In our patient with the IKBKG c.1167dup (ZF-truncating) mutation, NEMO cannot bind linear ubiquitin chains, leading to defective IKK activation and reduced NF-κB-dependent transcription. This impairs B-cell differentiation and class-switching (explaining low IgG) and alters T-cell homeostasis (contributing to decreased CD8+ T cells and increased B-cell proportion). Moreover, loss of NEMO disrupts the deubiquitination feedback loop, further aggravating immune dysregulation and promoting intestinal epithelial necroptosis, which underlies the patient’s early-onset colitis.

The patient presented at two months of age with chronic diarrhea, mucoid bloody stools and fluctuating leukocytosis. Physical examination revealed sparse hair, reduced sweating, and a non-pruritic rash-findings consistent with ectodermal dysplasia. Most patients with mutations in the NEMO gene subtype typically present with infections caused by pyogenic bacteria or mycobacteria (66), but the patient described in this report was infected with Pneumocystis. This pattern of infection is associated with the pathological effects that zinc finger (ZF) domain mutations may induce; such mutations disrupt the CD40L-mediated degradation of the NF-κB inhibitor IκB-α, leading to defects in immunoglobulin class switching, and are also associated with T-cell defects (26, 67). Most patients with *NEMO* deficiency exhibit reduced IgG and impaired polysaccharide antibody responses (22), These manifestations, together with hypogammaglobulinaemia, decreased CD8+T cells, increased B cells and elevated IL-17, indicate immune dysregulation. Elevated IL-17 levels may indicate Th17 pathway activation, contributing to mucosal inflammation and increased susceptibility to fungal infections (68). The global survival rate for NEMO patients following allogeneic hematopoetic cell transplantation in children is 74% (69). For patients with NEMO deficiency and severely impaired T-cell function, early HSCT can be life-saving. Therefore, the child underwent HSCT (22). The c.1167dup (p.Glu390ArgfsTer5) frameshift mutation identified in this patient results in a truncated NEMO protein lacking the C-terminal zinc finger (ZF) domain, thereby causing loss of function. This variant has been documented as one of the most prevalent pathogenic IKBKG mutations. Mutations affecting the ZF domain have been demonstrated to contribute to phenotypic diversity, with a wide range of variants having been identified as causative agents of various diseases. For instance, severe mutations such as X420W have been shown to lead to OL-EDA-ID, a condition that can be life-threatening in severe cases (11). As demonstrated in **Table 2**, a comprehensive review of the extant literature reveals a total of 12 previously reported cases (references in **Table 2**, plus the present case) that carry the identical c.1167dup frameshift mutation. Among these, all 12 exhibited immunodeficiency, 11 had ectodermal dysplasia features, and 7 presented with gastrointestinal involvement (chronic diarrhea, colitis, or bloody stools). It is noteworthy that the onset of colitis in the present case occurred at 2 months of age, which is earlier than most reported cases. Immunological phenotypes were identified, encompassing decreased IgG, variable T-cell subset abnormalities, and elevated B-cell counts in a number of patients. This comparison highlights the presence of phenotypic heterogeneity, i.e. the occurrence of different manifestations of the same disease, even among patients carrying the same IKBKG null mutation (2). This genotype-Phenotype Mismatch may result from a number of different mechanisms. Firstly, it is important to note that NF-κB signaling is pleiotropic. This indicates that the same loss-of-function mutation can yield significantly divergent consequences, contingent on the specific cell type and the inflammatory contex^t^ (70). However, it is also relevant to the mosaic expression of IKBG as a consequence of random X inactivation in heterozygous females (71). Furthermore, the modulation of environmental factors (such as differences in microbial exposure, gut microbiota composition, or concurrent infections) is likely to regulate the final clinical outcome (72).

Relevant mouse models have demonstrated that the activation of tumor necrosis factor (TNF) receptor 1 inhibits cell death downstream of the *NEMO/IKKα/IKKβ* complex, resulting in increased apoptosis in epithelial cells. The intestinal epithelium plays a key role in maintaining intestinal immune homeostasis. This leads to reduced expression of antimicrobial peptides, allowing bacteria to translocate into the mucosa. These bacteria then activate the innate immune response through pathways such as Toll-like receptors, which exacerbates intestinal damage and ultimately causes inflammatory bowel disease(IBD) (73, 74). Indeed, approximately 20% of male patients with *NEMO* deficiency develop intestinal inflammation, frequently accompanied by refractory diarrhea and/or growth failure (2). Gastrointestinal involvement is a salient yet often overlooked feature of *NEMO* deficiency. In the patient described here, the early onset of bloody stools and persistent occult blood suggest intestinal inflammation. Therefore, clinicians should consider X-linked ectodermal dysplasia with immunodeficiency in infants presenting with chronic diarrhea or colitis that is unexplained, especially if it is accompanied by ectodermal features or recurrent infections. This case further expands the clinical phenotype associated with this recurrent variant, emphasizing that early-onset colitis can be prominent features.

Treatment options include HSCT, antimicrobials and immunoglobulin replacement. HSCT is curative for the immunodeficiency, but not all manifestations are reverse, despecially in children with inflammatory bowel disease. Early HSCT improves prognosis, but mortality remains high without transplantation (69). Many untreated patients die from infections before one year of age, but early diagnosis and HSCT offer a chance for survival (10). There is still no cure, highlighting the urgent need for new treatments.

The study reports an EDA-ID case in a child caused by the c.1167dup variant in the *IKBKG* gene. It summarizes the clinical features, genetic diagnosis, and treatment process, while also analyzing the genotype-phenotype relationship. This case expands the clinical spectrum of the *IKBKG* c.1167dup mutation, particularly regarding severe gastrointestinal inflammation. Clinicians should remain vigilant for signs of ectodermal dysplasia and immunological abnormalities in early childhood.

## Data Availability

All relevant data is contained within the article: The original contributions presented in the study are included in the article/supplementary material, further inquiries can be directed to the corresponding authors.
